# Guest Editorial

**Published:** 2015-02-09

**Authors:** Srinivas Namineni

**Affiliations:** Professor and Head, Pioneer of Conscious Sedation Dentistry in India Course Director, Certificate Course in Sedation, Department of Pediatric Dentistry, Sri Sai Dental College, Vikarabad, Hyderabad, Andhra Pradesh

## Abstract

At the start of this New Year, I would like to draw attention of pediatric dental fraternity at large toward Raven Maria Blanco Foundation, United States. This is a foundation established by parents of a young girl called Maria, who lost her life in pediatric dental Office, which was not prepared to handle an emergency arising from a procedural sedation for dental treatment. This is very heartening for any parent. It was so heart rending that Grammy award singer Michael Crawford popularly known as ‘Magnedo7’ penned a song called Raven’s Song in her memory. This brings the focus on two important issues: sedation and emergency preparedness in our practices.^1^

**Wish you all a very Happy New Year and I hope all of you enjoyed a fantastic festive season!**

At the start of this New Year, I would like to draw attention of pediatric dental fraternity at large toward Raven Maria Blanco Foundation, United States. This is a foundation established by parents of a young girl called Maria, who lost her life in pediatric dental Office, which was not prepared to handle an emergency arising from a procedural sedation for dental treatment. This is very heartening for any parent. It was so heart rending that Grammy award singer Michael Crawford popularly known as ‘Magnedo7’ penned a song called Raven’s Song in her memory. This brings the focus on two important issues: sedation and emergency preparedness in our practices.^1^

**Figure:**
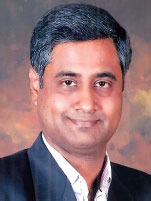


Procedural sedation in pediatric dentistry is here to stay. Unlike general anesthesia, sedation is always a controversial issue, when it is considered outside an operation theater and all the more when performed by nonanesthesiologists.^2^ The debate on this goes on across the globe. Many countries evolved with guidelines to govern the use of sedation in dental practice. Though our country woke up little late, efforts are on in this direction and we are all glad about it.

Among all drugs and routes of sedation used in pediatric dentistry, nitrous oxide-oxygen inhalation sedation (NOIS) has got an impeccable track record over 80 years with nil mortality!^3^ But the safety of it lies in its exclusive use without combining with any other drug and training in administration. Developing countries like ours need to take a fresh look at NOIS as it reduces the burden on resources and manpower due to its simplicity. Regulatory authority: Dental Council of India should give a serious thought on pushing guidelines on dental sedation, as the use of sedation is going to be inevitable because of increasing demand from the parents and children for pain-free dentistry.^4^

Accident is an ACCIDENT when it happens for the first time and only ignorance will let it happen second time. Emergencies arising from accidents during dental practice in pediatric population are not rare.^5^ Studies from around the developing world prove that only less than half of dentists are confdent to handle emergencies and not much data are available from this country.^6^ One need not learn from an emergency, rather be prepared to face one, as it happens. Because accidents do not come with a warning! Mainstream curriculum in dentistry does not seem to have addressed emergency preparedness skills effectively. Training in appropriate skills like BLS, ACLS and PALS should be made mandatory. Dental institutes should provide forums and facilities for the stakeholders to learn and upgrade emergency skills. Fraternity from their side need to know the responsibilities in safeguarding the welfare of the patients.

**Let us put our hearts into making lives of children healthy and happy.**

                                                                                                                               **Srinivas Namineni**

                                                                                     Pioneer of Conscious Sedation Dentistry in India

                                                                                       Course Director, Certificate Course in Sedation

                                                                                                                               Professor and Head

                                                                                                            Department of Pediatric Dentistry

                                                                                                            Sri Sai Dental College, Vikarabad

                                                                                                                    Hyderabad, Andhra Pradesh
